# The Chemical Master Equation Approach to Nonequilibrium Steady-State of Open Biochemical Systems: Linear Single-Molecule Enzyme Kinetics and Nonlinear Biochemical Reaction Networks

**DOI:** 10.3390/ijms11093472

**Published:** 2010-09-20

**Authors:** Hong Qian, Lisa M. Bishop

**Affiliations:** Department of Applied Mathematics, University of Washington, Seattle, WA 98195, USA

**Keywords:** chemical kinetics, chemical master equation, stochastic dynamics, biochemical reaction, systems biology

## Abstract

We develop the stochastic, chemical master equation as a unifying approach to the dynamics of biochemical reaction systems in a mesoscopic volume under a living environment. A living environment provides a continuous chemical energy input that sustains the reaction system in a nonequilibrium steady state with concentration fluctuations. We discuss the linear, unimolecular single-molecule enzyme kinetics, phosphorylation-dephosphorylation cycle (PdPC) with bistability, and network exhibiting oscillations. Emphasis is paid to the comparison between the stochastic dynamics and the prediction based on the traditional approach based on the Law of Mass Action. We introduce the difference between nonlinear bistability and stochastic bistability, the latter has no deterministic counterpart. For systems with nonlinear bistability, there are three different time scales: (*a*) individual biochemical reactions, (*b*) nonlinear network dynamics approaching to attractors, and (*c*) cellular evolution. For mesoscopic systems with size of a living cell, dynamics in (*a*) and (*c*) are stochastic while that with (*b*) is dominantly deterministic. Both (*b*) and (*c*) are emergent properties of a dynamic biochemical network; We suggest that the (*c*) is most relevant to major cellular biochemical processes such as epi-genetic regulation, apoptosis, and cancer immunoediting. The cellular evolution proceeds with transitions among the attractors of (*b*) in a “punctuated equilibrium” manner.

## 1. Introduction

Quantitative modelling in terms of mathematical equations is the foundation of modern physical sciences. If one deals with mechanical motions or electromagentic issues of daily lives, he/she starts with Newton’s Second Law or Maxwell’s equations, respectively. For work on the subatomic and molecular level, we have the quantum mechanics of Heisenberg and Schrödinger for the small things, and Gibbs’ statistical mechanics for large collections of particles. The last theory on the list, Gibbs’ statistical thermodynamics, has been the foundation of molecular science [1]. Its applications to biological macromolecules have laid the groundwork for molecular biology [2,3].

However, it has long been recognized that Gibbs’ theory can not be applied to a system outside chemical equilibrium. In this case, and when the deviations from an equilibrium are linear, Onsager’s theory provides the unifying approach known as linear irreversible thermodynamics. However, cellular biologists have long been aware of that most living processes are not near an equilibrium, but far from it. This begs an answer to the question: What is the theory one should use in modelling a biochemical reaction system in its living environment?

### 1.1. The Chemical Master Equation (CME)

Both Gibbs’ and Onsager’s work have pointed to a new type of mathematics: random variables and stochastic processes. Gibbs’ thermodynamic quantities with thermal fluctuations are random variables, and Onsager has used extensively Gaussian-Markov processes to describe the dynamics near an equilibrium [4,5]. This approach can be traced back to the earlier work of Einstein on Brownian motion and Smoluchowski on diffusion.

Quantitative modelling in chemical engineering has been based on the Law of Mass Action [6]. The applications of this theory to enzyme kinetics gives rise to the entire kinetic modelling of biochemical reactions for individual enzymes [7] and for enzymatic reaction systems [8]. However, the theory based on the Law of Mass Action considers no fluctuations so it sits in an odd position with respect to the more general theories of Gibbs and Onsager. It does have its strength though, it is a fully dynamic theory, while the Gibbs’ is not, and it can be applied to system beyond Onsager’s linear irreversibility.

Is there a theory which can embody all the above mentioned theories? It is clear that such a theory, even very imperfect, can provide great insights into the working of biochemical reaction systems in their living environment. While a consensus has not been reached, the recent rapid rise of applications of the Gillespie algorithm seems to suggest an interesting possibility.

It might be a surprise to some, but the Gillespie algorithm (GA) *is really an equation* that perscribes the dynamic trajectory of a stochastic process. Mathematical equations can come in many different forms: differential equations for continuous variables changing with time, stochastic differential equations (SDE) for the trajectories of continuous random variables changing with time, and the GA is simply the equation for the trajectory of discrete random variables changing with time. For a random variable changing with time one can either characterize it by its stochastic trajectories, as by the SDE and the GA, or one can characterize its probability distribution as a function of time. The corresponding equation for the SDE is the well-known Fokker-Planck equation, and the corresponding equation for the GA is called the *chemical master equation* (CME).

It has been mathematically shown that the CME approach is the mesoscopic version of the Law of Mass Action [8]. It is not a competing theory to the Law of Mass Action, rather, it extends the latter to the mesoscopic chemistry and biochemistry.

We do not expect our readers to have a background in the CME. For a quick introduction see Appendix. In particular, one should learn to draw the *chemical master equation graph*. See Chapter 11 of [8] and a more recent [9] for more on the CME. Discussions on the GA can be found in [10].

In this article, we shall follow the CME approach to study biochemical reaction networks. We are particularly interested in such systems situated in a “living envirnment”. It turns out, one of the precise defining characteristics of the environment is the amount of chemical energy pumped into the system – similar to the battery in a radio.

### 1.2. Nonequilibrium Steady State (NESS)

While the CME approach is a new methodological advance in modelling open (driven) biochemical systems, a new concept also arises from recent studies on open (driven) biochemical systems: the *nonequilibrium steady state* (NESS) as a mathematical abstraction of biochemical homeostasis. In terms of the CME approach to mesoscopic chemical and biochemical reaction systems there are three, and only three, types of dynamics [11]:

1. Equilibrium state with fluctuations which is well-understood according to Boltzmann’s law, and the theories of Gibbs, Einstein, and Onsager.
2. Time-dependent, transient processes in which systems are changing with time. In the past, this type of problems is often called “nonequilibrium problems”. As all experimentalists and computational modellers know, time-dependent kinetic experiments are very difficult to perform, and time-dependent equations are very difficult to analyze.
3. Nonequilibrium steady state: The system is no longer changing with time in a statistical sense, *i.e.*, all the probability distributions are stationary; nevertheless, the system is not at equilibrium. The systems fluctuate, but the fluctuations do not obey Boltzmann’s law. Such a system only eixsts when it is driven by a sustained chemical energy input. Complex deterministic dynamics discussed in the past, such as chemical bistability and oscillations, are all macroscopic limit of such systems.

To a first-order approximation, one can represent a biochemical cell or a subcellular network in homeostasis as a NESS. This is the theory being put forward by I. Prigogine, G. Nicolis and their Brussels group [12]. The NESS theory has recently gone through major development in terms of the fluctuation theorem in statistical physics, especially the stochastic version of J. Kurchan, J.L. Lebowitz and H. Spohn [13,14], and irreversible Markov processes in mathematics [15](A deep mathematical result shows that the arrow of time is a sufficient and necessary condition for the positiveness of an appropriately defined entropy production rate.). The theory of NESS also has enjoyed great appreciation through works on molecular motors [16,17].

To have a better understanding of the nature of a NESS, we list three key characteristics of a system in equilibrium steady state: First, there is no flux in each and every reaction. This is known as the principle of detailed balance [18]. Second, the system is *time-reversible*. One can play a “recording tape” of the system backward and will find it is statistically equivalent; There is no arrow of time [15]. The logical conseqeunce of the above statements is that any process occurred in an equilibrium system will have equal probability to run backward. Hence nothing can be accomplished. There is no energy transduction, and there is no signal processing.

For more discussions on NESS and its applications to biochemical systems and modelling, the readers are referred to [19,20].

## 2. The Chemical Master Equation and Its Applications to Kinetics of Isolated Enzyme

Since enzyme kinetics is the workhorse of biochemical reaction networks, let us start with the CME approach to the standard Michaelis-Menten (MM) enzyme reaction scheme:

(1) $$E+S\overset{k_{1}}{\underset{k_{-1}}{⇌}}ES\overset{k_{2}}{\to }E+P$$

In the CME approach to chemical and biochemical kinetics, one no longer asks what are the concentrations of *E*, *S*, *ES* and *P*, but instead, what is the probability of the system having *m* number of *S* and *n* number of *ES: p*(*m, n, t*) = Pr{*N_S_*(*t*) = *m, N_ES_*(*t*) = *n*} where *N_S_*(*t*) and *N_ES_* are the non-negative integer valued random variables, the number of *S* and *ES*. As functions of time, both are stochastic processes.

Assuming that the total number of substrate and product molecules is *m*_0_ = *N_S_*(*t*)+*N_P_* (*t*)+*N_ES_*(*t*), and total number of enzyme molecules is *n*_0_ = *N_E_*(*t*) + *N_ES_*(*t*), we have the CME (See Section 4 for the details to obtain the equation.)

(2) $$\begin{array}{l} \frac{dp(m,n,t)}{dt}=-\left({\hat{k}}_{1}m(n_{0}-n)+{\hat{k}}_{-1}n+{\hat{k}}_{2}n\right)p(m,n,t)\\ +{\hat{k}}_{1}(m+1)(n_{0}-n+1)p(m+1,n-1,t)\\ +{\hat{k}}_{-1}(n+1)p(m-1,n+1,t)\;\;\;(0\leq m\leq m_{0},0\leq n\leq n_{0})\\ +{\hat{k}}_{2}(n+1)p(m,n+1,t) \end{array}$$

There are three reactions in the kinetic scheme (1), hence there are six terms, three positive and three negative, in the CME (2). Note that the *k̂*’s in the above equation are related, but not the same as the rate constants *k*’s in Equation (1). The latter is concentration based, and the former is number based:

(3) $${\hat{k}}_{1}=\frac{k_{1}}{V},\;{\hat{k}}_{-1}=k_{-1},\;{\hat{k}}_{2}=k_{2}$$

### 2.1. Quasi-stationary approximation of the Michaelis-Menten enzyme kinetics

One of the most important results in deterministic enzyme kinetic theory is the quasi-steady state approximation leading to the well-known Michaelis-Menten equation for the production of the product in Equation (1):

(4) $$\frac{d[S]}{dt}=-\frac{d[P]}{dt}=-\frac{k_{1}k_{2}[S]E_{t}}{k_{-1}+k_{2}+k_{1}[S]}$$

where *E_t_* is the total enzyme concentration. We shall now carry out a parallel analysis for the CME (2).

As pointed out by Kepler and Elston [21], Rao and Arkin [22], and by Qian [16], the quasi-stationary approximation is best cast in terms of the *conditional probability*. If the dynamics in Equation (2) is such that the changes in *n* can reach stationarity first, due to *n*_0_ << *m*_0_, then one can first solve the problem of steady state conditional distribution *p*(*n*|*m*), then using this to solve the *p*(*m, t*). This is done as follows.

In the first step, on a fast time scale for fixed *m*, the Equation (2) can be written as

(5) $$\begin{array}{l} \frac{dp(n,t|m)}{dt}=-\left({\hat{k}}_{1}m(n_{0}-n)+{\hat{k}}_{-1}n+{\hat{k}}_{2}n\right)p(n,t|m)\\ +{\hat{k}}_{1}(m+1)(n_{0}-n+1)p(n-1,t|m)\\ +({\hat{k}}_{-1}+{\hat{k}}_{2})(n+1)p(n+1,t|m)\;\;\;(0\leq m\leq m_{0},0\leq n\leq n_{0}) \end{array}$$

Here we assumed *m*−1 ≈ *m*. This immdiate gives us the conditional stationary state distribution for *n*:

(6) $$p^{ss}(n|m)=\frac{n_{0}!}{n!(n_{0}-n)!}\frac{{\left({\hat{k}}_{-1}+{\hat{k}}_{2}\right)}^{n_{0}-n}{\left({\hat{k}}_{1}m\right)}^{n}}{{\left({\hat{k}}_{-1}+{\hat{k}}_{2}+{\hat{k}}_{1}m\right)}^{n_{0}}}\;\;\;(0\leq n\leq n_{0})$$

which yields a mean value for *n*, with given *m*:

(7) $$\langle n\rangle (m)=\sum_{n=0}^{n_{0}}{np}^{ss}(n|m)=\frac{{\hat{k}}_{1}{mn}_{0}}{{\hat{k}}_{-1}+{\hat{k}}_{2}+{\hat{k}}_{1}m}$$

This result agrees exactly with the deterministic model.

Now the second step, let us sum over all the *n* for the Equation (2). With

$$\sum_{n}p(m,n,t)=p(m,t)\;\;\;\text{and }\;\;p(m,n,t)\approx p(m,t)p^{ss}(n|m)$$

we have

(8) $$\begin{array}{l} \frac{dp(m,t)}{dt}=-\left({\hat{k}}_{1}m(n_{0}-\langle n\rangle (m))+{\hat{k}}_{-1}\langle n\rangle (m)\right)p(m,t)\\ +{\hat{k}}_{1}(m+1)(n_{0}-\langle n\rangle (m+1))p(m+1,t)\\ +{\hat{k}}_{-1}\langle n\rangle (m-1)p(m-1,t)\;\;\;(0\leq m\leq m_{0},0\leq n\leq n_{0}) \end{array}$$

**Figure 1:**
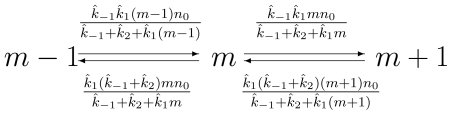
Through quasi-statioanry approximation, the CME in Equation (2), represented by the two-dimensional graph in Figure 6, is reduced to the one-dimensional system shown here. The corresponding master equation is shown in Equation (8).

Equation (8) is the result of a quasi-statioanry approximation. It should be compared with the deterministic equation (4). It can be graphically represented as in Figure 1.

We see that at any given time, *S* can increase or decrease by one. The decrease of one *S* is from either *ES* → *E* + *P* or *ES* → *E* + *S*, with probability $\frac{{\hat{k}}_{2}}{{\hat{k}}_{-1}+{\hat{k}}_{2}}$ and $\frac{{\hat{k}}_{-1}}{{\hat{k}}_{-1}+{\hat{k}}_{2}}$, respectively. Hence, the number of *P* will increase, at any given time, with the rate of

(9) $$\frac{{\hat{k}}_{1}{\hat{k}}_{2}{mn}_{0}}{{\hat{k}}_{-1}+{\hat{k}}_{2}+{\hat{k}}_{1}m}$$

This is exactly the CME version of Equation (4) [22]. The result shows that the waiting time between consecutive arrivial of *P* is exponentially distributed. This surprising result can be best understood from a mathematical theorem on the superposition of *N* identical, independent renewal processes [23]. For a more mathematical discussion on the subject, see [24].

Even when the number of enzymes is not large, the product arrival time distribution contains no information more than the traditional Michaelis-Menten rate constant. However, this is not the case if there is truly only a single enzyme. This will be discussed below.

### 2.2. Single-Molecule Michaelis-Menten Enzyme Kinetics

Now in Equation (2), let us consider *n*_0_ = 1. Then the equation is reduced to

(10a) $$\frac{dp(m,0,t)}{dt}=-{\hat{k}}_{1}mp(m,0)+{\hat{k}}_{-1}p(m-1,1)+{\hat{k}}_{2}p(m,1)$$

(10b) $$\frac{dp(m,1,t)}{dt}=-({\hat{k}}_{-1}+{\hat{k}}_{2})p(m,1)+{\hat{k}}_{1}(m+1)p(m+1,0)$$

This is the CME for a single molecule enzyme kinetics according to the MM in Equation (1). Often, one is only interested in the conformational states of the enzyme:

$$p_{E}(t)=\sum_{m=0}^{m_{0}}p(m,0,t),\;\;\;p_{ES}(t)=\sum_{m=0}^{m_{0}}p(m,1,t)$$

Carry out the summation on the both sides of Equation (10), we have

(11a) $$\frac{dp_{E}(t)}{dt}=-{\hat{k}}_{1}\langle N_{S}\rangle p_{E}(t)+({\hat{k}}_{-1}+{\hat{k}}_{2})p_{ES}(t)$$

(11b) $$\frac{dp_{ES}(t)}{dt}=-({\hat{k}}_{-1}+{\hat{k}}_{2})p_{ES}(t)+{\hat{k}}_{1}\langle N_{S}\rangle p_{E}(t)$$

where

(12) $$\langle N_{S}\rangle =\frac{\sum_{m=0}^{m_{0}}mp(m,0,t)}{\sum_{m=0}^{m_{0}}p(m,0,t)},\;\;\;{\hat{k}}_{1}\langle N_{S}\rangle =k_{1}c_{S}$$

*c_S_* is the concentration of *S*. Equation (11) is the stochastic model for a single enzyme molecule dynamics.

The steady state probability for the single enzyme can be easily obtained from Equation (11):

(13) $$p_{E}^{ss}=\frac{k_{-1}+k_{2}}{k_{1}c_{S}+k_{-1}+k_{2}},\;\;\;p_{ES}^{ss}=\frac{k_{1}c_{S}}{k_{1}c_{S}+k_{-1}+k_{2}}$$

Then the steady state single enzyme turnover flux is

(14) $$J^{ss}=k_{2}p_{ES}^{ss}=\frac{k_{1}k_{2}c_{S}}{k_{1}c_{S}+k_{-1}+k_{2}}=\frac{V_{max}c_{S}}{K_{M}+c_{S}}$$

with $k_{M}=\frac{K_{-1}+k_{2}}{k_{1}}$ and *V_max_* = *k*_2_. The last expression is precisely the Michaelis-Menten formula.

The single enzyme steady-state flux *J^ss^* is exactly the inverse of the mean time duration between two product arrivials. The *time perspective* is natural for single-molecule measurements on enzyme turnover [23]. Not only one can obtain the mean time duration, one can also, according to the stochastic model in Equation (11), compute the probability density function (pdf) of the time duration between two product arrivals. The pdf is exponentially distributed if there is a single *rate-limiting step* with in the enzyme cycle. In general, however, it is not exponentially distributed.

### 2.3. Driven Enzyme Kinetics

We now consider an enzyme kinetic scheme that is a little more complex than that in Equation (1):

(15) $$E+S\overset{k_{1}}{\underset{k_{-1}}{⇌}}ES\overset{k_{2}}{\underset{k_{-2}}{⇌}}EP\overset{k_{3}}{\underset{k_{-3}}{⇌}}E+P$$

With concentrations *c_S_* and *c_P_* for *S* and *P* being constant, as many cases in a living cell under homeostasis, we have the steady state single enzyme turnover flux

(16) $$J^{ss}=\frac{k_{1}k_{2}k_{3}c_{S}-k_{-1}k_{-2}k_{-3}c_{P}}{k_{2}k_{3}+k_{-2}k_{-1}+k_{3}k_{-1}+(k_{1}k_{2}+k_{1}k_{-2}+k_{3}k_{1})c_{S}+(k_{-1}k_{-3}+k_{2}k_{-3}+k_{-3}k_{-2})c_{P}}$$

The origin of this flux is the non-equilibrium between the chemical potentials of *S* and *P*:

(17) $$\Delta \mu =\mu_{S}-\mu_{P}=\mu_{S}^{o}+k_{B}T\text{ln}c_{S}-\mu_{P}^{o}-k_{B}T\text{ln}c_{P}=k_{B}T\text{ln}\frac{k_{1}k_{2}k_{3}c_{S}}{k_{-1}k_{-2}k_{-3}c_{P}}$$

We see that when Δ*μ >* 0, *J^ss^* *>* 0, when Δ*μ <* 0, *J^ss^* *<* 0, and when Δ*μ* = 0, *J^ss^* = 0. In fact, the product Δ*μ* × *J^ss^* is the amount of chemical energy input to the single enzyme. If the enzyme does not do any mechanical work such as a motor protein, then all this energy becomes heat and dissipated into the aqueous solution in which the enzymatic reaction occurs.

Let us see an example of *J^ss^* as function of Δ*μ*, for *k_i_* = λ, *k*_−_*_i_* = 1, *i* = 1, 2, 3, and *c_P_* = 1 while changing the *c_S_*:

(18) $$J^{ss}=\frac{\lambda^{3}c_{S}-1}{(3+2\lambda +\lambda^{2})+(1+2\lambda )\lambda c_{S}}=\frac{\lambda^{2}(e^{\Delta \mu /k_{B}T}-1)}{(3+2\lambda +\lambda^{2})\lambda^{2}+(1+2\lambda )e^{\Delta \mu /k_{B}T}}$$

**Figure 2:**
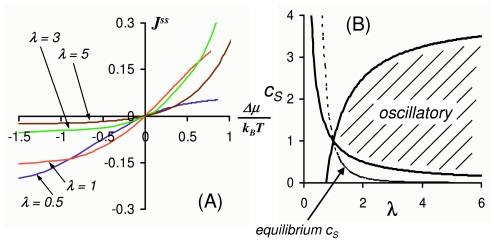
Simple enzyme kinetic system in Equation (15) exhibits nonlinear flux (*J^ss^*)-potential (Δ*μ*) realtion and oscillatory behavior. The parameters used: *k*_1_ = *k*_2_ = *k*_3_ = λ, *k*_−1_ = *k*_−2_ = *k*_−3_ = 1, and *c_P_* = 1. **(A)** The steady state flux *J^ss^* is given in Equation (18), and the chemical potential Δ*μ* is given by Equation (17): Δ*μ*/*k_B_T* = ln(λ^3^*c_S_*). Each curve is obtained by fixed λ, as indicated, with varying *c_S_*. **(B)** The region of parameter values for λ and *c_S_* in which there are complex eigenvalues is given in Equation (20). The dashed line represents the equilibrium *c_S_^eq^*, which is outside the oscillatory region.

Figure 2A shows several curves. We see that their relationship is not linear over the entire range of Δ*μ*. Only when the Δ*μ* is very small, there is a linear region *J^ss^* = Δ*μ*/*k_B_T*(λ^4^ +2λ^3^ +3λ^2^ +2λ+1). This is the region where Onsager’s theory applies. In fact, the linear coefficient between Δ*μ* and *J^ss^* is precisely the *one-way flux* in the equilibrium. To show this, we note from Equations (16) and (17) that *J^ss^* = *J*^+^ − *J*^−^ and Δ*μ* = *k_B_T* ln(*J*^+^*/J*^−^). Then, when *J^ss^* *<<J*^−^, we have

(19) $$J^{ss}=J^{+}-J^{-}=J^{-}(e^{\Delta \mu /k_{B}T}-1)=\frac{J^{-}}{k_{B}T}\Delta \mu$$

Note that in equilibrium, *J*^+^ = *J*^−^. The last equation is known as *detailed balance*, which plays a central role in Onsager’s theory.

Therefore, the simple enzyme kinetics is not in the region with linear irreversibility. Onsager’s theory does not apply. Interestingly, we also note that the nonlinear curves in Figure 2A very much resemble the curret-voltage characteristics of a semiconductant diode. It will be interesting to further explore the similarities between driven biochemical and electronic systems [25].

### 2.4. Far from Equilibrium and Enzyme Oscillations

In fact, one of the most important results in Onsager’s linear theory is the *reciprocal relations* [26,27] which, based on the principle of detailed balance, dictates certain symmetry in the kinetics. One of the consequences of this symmetry is that chemical kinetics near equilibrium can not oscillate. Oscillatory kinetics are associated with the complex eigenvalues of the kinetics system. For the scheme in Equation (15), the imaginary component of its eigenvalues is $\sqrt{4\Delta -\Lambda^{2}}$, where

$$\begin{array}{c} \Lambda =k_{1}c_{S}+k_{2}+k_{3}+k_{-1}+k_{-2}+k_{-3}c_{P}\\ \Delta =k_{2}k_{3}+k_{-2}k_{-1}+k_{3}k_{-1}+(k_{1}k_{2}+k_{1}k_{-2}+k_{3}k_{1})c_{S}+(k_{-1}k_{-3}+k_{2}k_{-3}+k_{-3}k_{-2})c_{P} \end{array}$$

To see an example, let us again consider *k*_1_ = *k*_2_ = *k*_3_ = λ, and *k*_−1_ = *k*_−2_ = *k*_−3_ = 1, and *c_P_* = 1. Then

$$\sqrt{4\Delta -\Lambda^{2}}=\sqrt{(3-4\lambda )+2\lambda (2\lambda -1)c_{S}-\lambda^{2}c_{S}^{2}}$$

The oscillations exist for

(20) $$\frac{1}{\lambda }<c_{S}<\frac{4\lambda -3}{\lambda },\;\text{when }\lambda >1\;\text{and }\frac{4\lambda -3}{\lambda }<c_{S}<\frac{1}{\lambda }\;\text{when }\lambda <1$$

We see from Figure 2B that in general the *c_S_* has to be sufficiently far away from its equilibrium value in order to have the oscillation. Chemical and biochemical oscillation is a far-from-equilibrium phenomenon [28,29].

## 3. The CME Approach to Nonlinear Biochemical Networks in Living Environment

In a living cell, one of the most important, small biochemical regulatory networks is the phosphorylation-dephosphorylation cycle (PdPC) of an enzyme, first discovered by E.H. Fischer and E.G. Krebs in 1950s. It consists of only three players: a substrate enzyme, a kinase and a phosphatase. The phosphorylation of the substrate protein *E*, $ATP+E\overset{k_{1}}{\to }ADP+E^{*}$, is catalyzed by the kinase *K*, and its dephosphorylation $E^{*}\overset{k_{2}}{\to }E+Pi$ is catalyzed by the phosphatase *P*. Even though it is traditionally called *reversible chemical modification*, one should note that these two steps are different chemical reactions. In fact, a kinase should also catalyze the reaction $ADP+E^{*}\overset{k_{-1}}{\to }ATP+E$, as should the phosphatase for $E+Pi\overset{k_{-2}}{\to }E^{*}$. These latter two reactions are simply too slow, even in the presense of the respective enzymes, to be noticed, but they definitely can not be zero. The proof is that the complete of a PdPC is the hydrolysis of a single *ATP* to *ADP* + *Pi*. This reaction has an equilibrium constant of 4.9 × 10^5^ M [30], which means

(21) $$\frac{k_{1}k_{2}}{k_{-1}k_{-2}}=K_{ATP}=4.9\times 10^{5}$$

### 3.1. Phosphorylation-Dephosphorylation Cycle with Autocatalysis: A Positive Feedback Loop

Many kinase itself can exist in two different forms: an inactive state and an active state. Furthermore, the conversion from the former to the latter involves the binding of the E*, sometime one, sometime two. Hence, we have [31–33]:

(22) $$K+\chi E^{*}\overset{K_{a}}{⇌}K^{‡}$$

where χ = 1, 2. We shall call χ = 1 first-order autocatalysis and χ = 2 second-order autocatalysis. Therefore, if the conversion is rapid, then the active kinase concentration is [*K*^‡^] = *K_a_*[*K*][E*]^χ^. Now combining the reaction in Equation (22) with the PdPC, such a mechanism is called autocatalysis: more E* is made, more *K*^‡^, which in turn to make more E*. Quantitatively, the rate of phosphorylation reaction catalyzed by the active kinase is:

(23) $$\frac{d[E^{*}]}{dt}=k_{1}[ATP][K^{‡}][E]=k_{1}[ATP]K_{a}[K]{\left[E^{*}\right]}^{\chi }[E]$$

where [*X*] denotes the concentration of biochemical species *X*. Note, however, that the same kinase *K*^‡^ also catalyzed the reverse reaction of the phosphorylation. Hence, to be more realistic, we have

(24) $$\begin{array}{l} \frac{d[E^{*}]}{dt}=K_{a}[K]{\left[E^{*}\right]}^{\chi }(k_{1}[ATP][E]-k_{-1}[ADP][E^{*}])\\ =\alpha {\left[E^{*}\right]}^{\chi }[E]-\beta {\left[E^{*}\right]}^{\chi +1} \end{array}$$

in which

$$\alpha =k_{1}K_{a}[ATP][K]\;\;\;\text{and }\;\;\beta =k_{-1}K_{a}[ADP][K]$$

contains the concentration of *ATP* and *ADP* respectively. Equation (24) is the kinetic equation for the phosphorylation reaction catalyzed by a kinase which is activated by binding χ number of E*.

**Figure 3:**
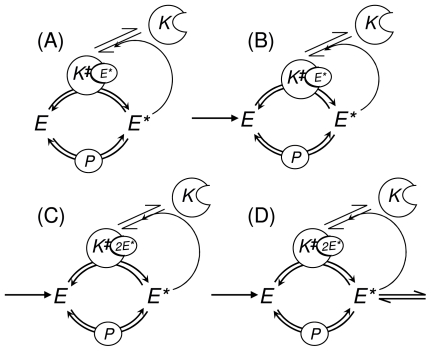
An assorted variations of the PdPC with autocatalytic feedback. The phosphorylation of the substrate *E* to E* is catalyzed by an active form of the kinase *K*^‡^, and the dephosphorylation is catalyzed by a phosphatase (*P*). The activation of the kinase involves the binding of*K* to E*. In **(A)** and **(B)** the autocatalysis is first order: *K*+E* ⇌ *K*^‡^; In **(C)** and **(D)**, it is second order: *K* + 2E* ⇌ *K*^‡^. The nonlinear feedback in the latter is stronger; thus they exhibit more pronounced nonlinear behavior: bistability and limit cycle oscillation.

Figure 3 shows four, with subtle differences, PdPC with such a positive feedback loop. Biochemical examples of this type of regulation are MPAK pathway [31], Src Family kinase pathway [32], and Rabaptin-5 mediated Rab5 activation in endocytosis [33]. We shall now establish the appropriate kinetic equations for each of these nonlinear biochemical networks.

### 3.2. Stochastic Bistability in PdPC with First-Order Autocatalysis

For the kinetic scheme in Figure 3A, we have χ = 1 and

(25) $$\frac{dx}{dt}=\alpha x(x_{t}-x)-\beta x^{2}-ɛx+\delta (x_{t}-x)$$

here we use *x* to denote the [E*], *x_t_* = [*E*] + [E*]. In the equation

$$ɛ=k_{2}[P]\;\;\;\text{and }\;\;\delta =k_{-2}[P][Pi]$$

represent the rates for the dephosphorylation and the rate for its reverse reaction, respectively. Both are catalyzed by the enzyme phosphatase *P*. For simplicity, we assume both kinase and phosphatase are operating in their linear region.

Bishop and Qian [34] have carefully studied Equation (25). While this model is very simple, the issues arise from the model are important, and have not been widely discussed. It is well-known, and as we shall discuss in Section 3.4, nonlinear open chemical and biochemical reaction systems can exhibit bistability, which plays a crucial role in cellular genetic [35] and signal regulations [20,36]. In fact, it has been argued that bistability is one of the key origins that generate complex dynamic behavior [37]. Bistable chemical reaction systems have been extensively studied in the past [38]. In fact, it is relatively easy to theoretically construct reaction schemes that show bistability and bifurcation. Since bistability mathematically means two stable and one unstable fixed points in the positive quardrant, it is easy to show, according to the Law of Mass Action, that a tri-molecular reaction (as a reduced mechanism for multisteps of bimolecular reactions) is necessay.

In [34], however, we discovered a simpler bi-molecular chemical reaction system that possibly exhibits “bistability”. The bistability is in quotation marks since the mechanism is very different from that in traditional nonlinear reactions. The system is modelled in terms of a CME, and the bistability and (saddle-node) bifurcation are purely stochastic phenomenon. They only occur in reaction systems with small volume and small number of molecules.

#### 3.2.1. Deterministic Kinetics of PdPC with First-Order Autocatalysis and Delayed Onset

Let *y* be the concentration ratio of *x*/*x_t_*, the fraction of the substrate enzyme in the phosphorylated state. Also introduce nondimensional variables and parameters

$$\tau =(\alpha +\beta )x_{t}t,\;a_{1}=\frac{\alpha x_{1}-ɛ-\delta }{(\alpha +\beta )x_{t}},\;a_{0}=\frac{\delta }{(\alpha +\beta )x_{t}}$$

then Equation (25) can be simplified as

(26) $$\frac{dy}{d\tau }=-y^{2}+a_{1}y+a_{0}$$

Let

(27) $$\lambda_{1,2}=\frac{a_{1}\pm \sqrt{a_{1}^{2}+4a_{0}}}{2}$$

and λ_1_ be the one of the two roots ∈ (0, 1). Since λ_1_λ_2_ = −*a*_0_ *<* 0, λ_2_ *<* 0

The solution to Equation (26) is

(28) $$x(\tau )=\frac{\lambda_{1}(x_{o}-\lambda_{2})-\lambda_{2}(x_{o}-\lambda_{1})e^{(-\lambda_{1}+\lambda_{2})\tau }}{(x_{o}-\lambda_{2})-(x_{o}-\lambda_{1})e^{(-\lambda_{1}+\lambda_{2})\tau }}$$

in which *x_o_* = *x*(0), *x*(∞) = λ_1_

If both phosphorylation and dephosphorylation reactions are irreversible, as usually assumed in cell biology (When considering kinetics, but not thermodynamics, this is indeed valid for large ATP hydrolysis free energy in a living cell), then the reaction is simplified to

(29) $$E^{*}+E\overset{a}{\to }E^{*}+E^{*},\;\;\;E^{*}\overset{ɛ}{\to }E$$

where *α* and *ɛ* are proportional the the kinase and phosphatase activity, respectively. The differential equation in Equation (25) is simplified to

(30) $$\frac{dy}{dt}=\alpha x_{t}y(1-y)-ɛy$$

Its steady state exhibits a transcritical bifurcation as a function of the activation signal, *θ* = *αx_t_*/*ɛ*:

(31) $$y=\{\begin{array}{cc} 0 & 0\leq \theta \leq 1\\ 1-\frac{1}{\theta } & \theta \geq 1 \end{array}$$

**Figure 4:**
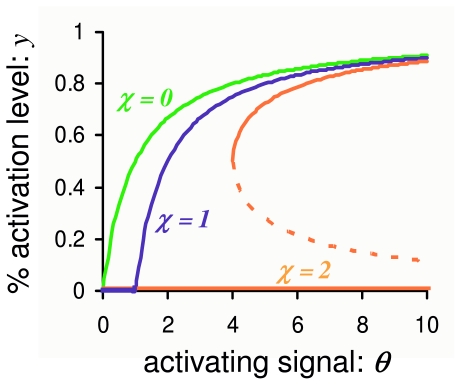
Activation curves of PdPC with or without autocatalytic phosphorylation *E* + χ E* → E* + χE* and dephosphorylation E* → *E*. Curve with χ = 0 is the standard hyperbolic activation without feeback: $y=\frac{\theta }{1+\theta }$. Curve with χ = 1 is for the PdPC with first-order autocatalysis, following Equation (31). It exhibits an extreme version of sigmoldal shape called *delayed onset*. Curve labelled χ = 2, following Equation (40), is for PdPC with second-order autocatalysis. It shows bistability when *θ >* 4, with the dotted branch being unstable.

Compared with the hypobolic activation curve $\frac{\theta }{1+\theta }$, Equation (31) exhibits “delayed onset” of activation [33,39]. See Figure 4. Note the curve (31) is an extreme version of a sigmoidal shape. It has a response coefficient of 9, and a Hill’s coefficient of 2. Recall that the response coefficient is defined as *θ*_0.9_/*θ*_0.1_, where *y*(*θ*_0.9_) = 0.9 and *y*(*θ*_0.1_) = 0.1.

It is interesting to point out that the curve in Equation (31), the delayed onset, can be obtained from a completely different mechanism for PdPC with multiple phosphorylation sites [40]. Assuming that there is a sequential phosphorylation of cites with rate *α* and dephosphorylation rate *β*, and there are totally *n* sites. The active form of the substrate enzyme requires full *n*-sites phosphorylation. Then

(32) $$y=\frac{{\hat{\theta }}^{n}}{1+\hat{\theta }+{\hat{\theta }}^{2}+\cdots {\hat{\theta }}^{n}}=\frac{{\hat{\theta }}^{n}(1-\hat{\theta })}{1-{\hat{\theta }}^{n+1}}$$

where *θ̂* = *α*/*β*. One can easily show that if *n* → ∞, the *y*(*θ̂*) will be precisely the one in Equation (31). Both mechanisms lead to the same mathematical expression of the activation curve.

#### 3.2.2. Stochastic Bistability and Bifurcation without Deterministic Counterpart

Consider the first order autocatalytic system from Equation (29), adding the appropriate reverse reactions such that the system can be considered in a thermodynamic framework, yields

(33) $$E^{*}+E\overset{k_{1}^{\prime }}{\underset{k_{-1}^{\prime }}{⇌}}E^{*}+E^{*},\;\;\;E^{*}\overset{k_{2}}{\underset{k_{-2}}{⇌}}E$$

The stochastic model of this system was studied in depth by Bishop and Qian, [34]. The appropriate CME, where *N*(*t*) is the random variable measuring the number of phosphorylated E* molecules and *N_t_* is the total number of kinase molecules, is

(34) $$\begin{array}{l} \frac{dp(n,t)}{dt}=-[k_{1}n(N_{t}-n)+k_{-1}n(n-1)+k_{2}n+k_{-2}(N_{t}-n)]p(n,t)\\ +[k_{1}(n-1)(N_{t}-n+1)+k_{-2}(N_{t}-n+1)]p(n-1,t)\\ +[k_{-1}(n+1)n+k_{2}(n+1)]p(n+1,t) \end{array}$$

where *k_±_*_1_ = *k*_±1_′/*V*.

**Figure 5:**
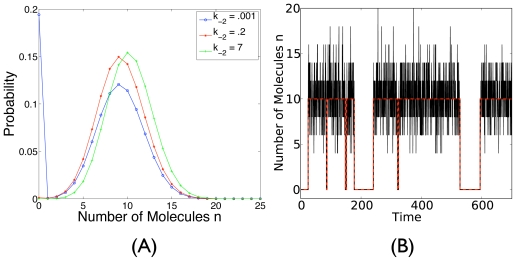
**(A)** The steady state distribution of the number of active kinase, N, from Equation 35. For certain parameter values the distribution is bimodal with the second peak appearing at zero. Parameter values are *k*_1_ = 5, *k*_−1_ = 10, *k*_2_ = 10, *N_t_* = 30 and *k*_−2_ varied. **(B)** Sample trajectory of the fluctuating E* in Equation 33 generated using the Gillespie Algorithm with parameter values, *k*_1_ = 5, *k*_−1_ = 10, *k*_2_ = 10, *k*_−2_ = 0.001, *N_t_* = 30. For each segment of nonzero fluctuations the average was taken and plotted (*dashed line*). Data taken from and figure redrawn based on [34].

Solving $\frac{dp(n,t)}{dt}=0$ leads to the steady state distribution

(35) $$p_{ss}(n)=C\prod_{j=0}^{n-1}\frac{\left(k_{1}j+k_{-2})(N_{t}-j\right)}{\left(k_{-1}j+k_{2})(j+1\right)}$$

where C is a normalization constant. For certain parameter regimes this distribution is bimodal where the bimodality appears as a sudden second peak at zero, Figure (5). This bimodal distribution is related to traditional deterministic dynamics by considering the peaks of the probability to correspond to stable steady states, and the troughs to correspond to unstable steady states. Figure (5B) shows how this unique instance of bi-molecular bistability is related to zero being almost an absorbing state.

Note that this bistability is a purely stochastic phenomenon; it has no deterministic counterpart. The deterministic model of the same bi-molecular system in Equation (30) and Figure (4) has only a weak (quadratic) nonlinearity and has no capacity bistability. Bishop and Qian showed that this stochastic bistability explains the more complex instance of the noise induced bistability first discovered in [41].

The extrema of Equation (35) can be conditioned on both the volume, *V*, and the energy, *γ*= (*k*_1_*k*_2_)/(*k*_−1_*k*_−2_), of the system. If we consider *V* to be the bifurcation parameter we can find that for 0 *< k*_−1_′/(*k*_1_′ *E_t_* −*k*_2_ −*k*_−2_) *< V < k*_2_/(*k*_−2_*E_t_*) the system is bistable. Letting *γ* be the bifurcation parameter we find *γ > k*2(*k*_−1_ + *k*2 + *k*_−2_)/(*N_t_k*_−1_*k*_−2_) with no upper bound, *i.e.*, a minimal energy input is necessary. These bounds with the parameters from Figure (5) clearly demonstrates that the stochastic bistability is dependent on having a sufficiently small volume and the presence of sufficiently large energy dissipation.

### 3.3. Keizer’s Paradox

For the kinetic scheme in Figure 3B, we again have χ = 1. However, we assume that there is a continuous biosynthesis and degradation for the *E* such that its concentration is sustained in the biochemical system, say at the value of *a*. Then, the kinetic equation for the dynamics of [E*] becomes

(36) $$\frac{dx}{dt}=\alpha ax-\beta x^{2}-ɛx+\delta a$$

When *δ* = 0, Equation (36) is the same equation for the generic chemical reaction scheme

(37) $$A+X\overset{\alpha }{\underset{\beta }{⇌}}2X,\;\;\;X\overset{ɛ}{\to }B$$

J. Keizer studied this model in [42] to illustrate a very interesting observation: While the deterministic kinetics of the system has a positive steady state, the steady state of its stochastic kinetics is zero. Vellela and Qian have studied this Keizer’s “paradox” [43]. It was shown that there are two very different time scales in the stochastic model: In a rather short time scale corresponding to the eigenvalues |λ_2_| and above, the system rapidly settles to a quasi-stationary distribution peaking at the deterministic positive steady state. However, in a much slower time scale corresponding to the eigenvalue |λ_1_|, the above probability distribution slowly decay to zero. For very large reaction system volume *V*, λ_1_ ~ −*e*^−^*^cV^* where *c* is a positive constant. Hence there is an exponentially slow decay process beyond the infinite time of the deterministic dynamics [44,45].

Keizer’s paradox and its resolution is the origin of all the multi-scale dynamics in the CME system with multi-stability. It is also clear it is intimately related to the stochastic bistability in Section 3.2.2 when the *k*_−2_, *i.e.*, *δ* in Equation (36), is very small but nonzero. *k*_−2_ controls the lifetime, *i.e.*, relative probability of the the zero state in Figure 5.

### 3.4. Schlögl’s Nonlinear Bistability and PdPC with Second-Order Autocatalysis

For the kinetic scheme in Figure 3C with second-order autocatalysis, we have χ = 2. We again assume that there is a continuous biosynthesis and degradation for the *E* such that its concentration is sustained in the biochemical system at a constant value of *a*. Then, the kinetic equation for the dynamics of *x* = [E*] becomes

(38) $$\frac{dx}{dt}=\alpha ax^{2}-\beta x^{3}-ɛx+\delta a$$

On the other hand, if we assume the rate of biosynthesis is negligible, and that both kinase and phosphatase catalyzed reactions are irreversbile, then we have the kinetics

(39) $$2E^{*}+E\overset{\alpha }{\to }3E^{*},\;\;\;E^{*}\overset{ɛ}{\to }E$$

Comparing this system with that in Equation (29), the difference is in the 2E* on the left-hand-side. The kinetic equation for the fraction of E*, $y=\frac{\left[E^{*}\right]}{E_{t}}$ where *E_t_* is the total amount of [*E*] + [E*]. is $\frac{dy}{dt}=\alpha E_{t}^{2}y^{2}(1-y)-ɛy$. The steady state exhibits a saddle-node bifurcation at $\theta =\frac{\alpha E_{t}^{2}}{ɛ}=4$:

(40) $$y=\{\begin{array}{cc} 0 & 0\leq \theta \leq 4\\ 0,\frac{\theta \pm \sqrt{\theta^{2}-4\theta }}{2\theta } & \theta \geq 4 \end{array}$$

See the orange curve in Figure 4.

Equation (38) is precisely the same kinetic equation, according to the Law of Mass Action, for the chemical reaction system

(41) $$A+2X\overset{\alpha }{\underset{\beta }{⇌}}3X,\;\;\;X\overset{ɛ}{\underset{\delta a/c_{B}}{⇌}}B$$

The system (41) is known as Schlögl’s model. It is the canonical example for nonlinear chemical bistability and bifurcation which has been studied for more than 30 years [46].

Qian and Reluga [47] have studied a system very similar to Equation (41) in terms of deterministic, nonlinear bifurcation theory. In particular, they established the important connection between the nonlinear bistability with nonequilibrium thermodynamics [48]. They have shown that if the concentrations of *A* and *B* are near equilibrium,

(42) $${\left(\frac{c_{B}}{a}\right)}^{eq}=\frac{c_{B}\alpha ɛ}{\beta \delta a},\;\;\;i.e.,\;\frac{\beta \delta }{\alpha ɛ}=\frac{k_{-1}k_{-2}[ADP][Pi]}{k_{1}k_{2}[ATP]}=1$$

then there would be no bistability. The last equation in (42) is precisely equivalent to free energy change of ATP hydrolysis being zero. It can be easily shown, see Section 4.1, with the equilibrium condition in Equation (42), the system has only a single, unique deterministic steady state. And also, in terms of its CME, a single peak in the equilibrium probability for the number of *X*. This result is much more general for all nonlinear chemical and biochemical reaction systems, not just limited to the simple reaction system in (41) [36,49,50].

Vellela and Qian [36] have recently studied the Schlögl system in great detail, with a nonequilibrium steady state perspective. In particular, it was shown that the nonlinear bistability is intimately related to nonequilibrium phase transitions in statistical physics [36,51,52]. Ge and Qian [45,51] further investigated the steady state distribution according to the CME and its relationship to the steady states according to the deterministic Law of Mass Action. They have shown that in the limit of system’s volume tends infinity, *i.e.*, the so called thermodynamic limit, the CME steady state(s) differ from that of deterministic model: A *Maxwell construction* like result is obtained: According to the CME, only one of the two determinsitic stable fixed point is the true global minimum, the other stable fixed point is only metastable. Hence in the thermodynamic limit, the global minimum has probability 1 while the metastable minimum has probability 0. However, the lifetime of the metastable state is infinitely long. Furthermore, using the mathematical tool of large deviation theory, [45] shows that the bistable CME system exhibits several key characteristics of nonequilibrium phase transition well-known in condensed matter physics.

### 3.5. Schnakenberg’s Oscillation

For the kinetic scheme in Figure 3D, we again have χ = 2, and we again assume that there is a continuous biosynthesis for the *E*. However, we now consider the dynamics of both [E*] and [*E*], denoted by *x* and *y*, respectively. Then, the kinetic equations becomes

(43a) $$\frac{dx}{dt}=\alpha yx^{2}-\beta x^{3}-ɛx+\delta y-\varphi x+\psi b$$

(43b) $$\frac{dy}{dt}=-\alpha yx^{2}+\beta x^{3}+ɛx-\delta y+\nu a$$

The system of Equations (43) is the same system for the kinetic scheme

(44) $$A\overset{\nu }{\to }Y,\;\;\;Y+2X\overset{\alpha }{\underset{\beta }{⇌}}3X,\;\;\;X\overset{ɛ}{\underset{\delta }{⇌}}Y\;\;\;X\overset{\varphi }{\underset{\psi }{⇌}}B$$

with [*A*] = 1 and [*B*] = 1. If the *β* = *ɛ* = *δ* = 0, then it becomes the celebrated Schnakenberg model which is well-known to exhibit periodic chemical oscillation. Qian *et al*. [53] first studied its stochastic dynamics in terms of the CME. Recently, Vellela and Qian [54] again have studied this system. In particular, they have introduced a novel mathematical concept of Poincaré-Hill cycle map (PHCM) to characterize the amplitude of rotational random walk. The PHCM combines the Poincaré map from nonlinear dynamic analysis [55] with the cycle kinetic analysis developed by T.L. Hill [56,57].

#### 3.5.1. Sel’kov-Goldbeter-Lefever’s Glycolytic Oscillator

So far, we have always assumed that the kinase catalysis is in its linear region, and avoided using Michaelis-Menten kinetic model for the kinase catalyzed phosphorylation. If we take the nonlinear Michaelis-Menten kinetics into account, interestingly, we discover that in this case, our model of PdPC with feedback in Figure 3D is mathematically closely related to a well-known metabolic oscillator: The Sel’kov-Goldbeter-Lefever model for glycolytic oscillation [58–60]:

(45) $$A\overset{\nu }{\to }Y,\;\;\;Y+K^{‡}\overset{\alpha_{1}}{\underset{\beta_{1}}{⇌}}YK^{‡}\overset{\alpha_{2}}{\to }X+K^{‡},\;\;\;K+2X\overset{\alpha_{3}}{\underset{\beta_{3}}{⇌}}K^{‡},\;\;\;X\overset{\varphi }{\to }B$$

In the glucolytic model, *X* and *Y* are ADP and ATP, *K* and *K*^‡^ are the inactive and activated from of phosphofructokinase-1. One can find a nice nonlinear analysis of the deterministic model based on the Law of Mass Action in [60], which shows limit-cycle oscillation. As far as we know, a stochastic analysis of this model has not been carried out.

## 4. Conclusions

Nonlinear chemical reactions are the molecular basis of cellular biological processes and functions. Complex biochemical reactions in terms of enzymes and macromolecular complexes form “biochemical networks” in cellular control, regulation, and signaling. One of the central tasks of cellular systems biology is to quantify and integrate experimental observations into mathematical models that first repreduce and ultimately predict laboratory measurements. This review provides an introduction of the biochemical modeling paradigm in terms of the chemical master equation (CME) and explores the dynamical possibilities of various biochemical networks by considering models of homogenous, *i.e.*, well-mixed, reaction systems with one and two dynamic variables. From mathematical modeling perspective, these are one- and two-dimensional system, the simplest to be fully explored with sufficient depth.

The chemical master equation is a comprehensive mathematical theory that quantitatively characterize chemical and biochemical reaction system dynamics [38,61]. Traditional chemical kinetics based on the Law of Mass Action, in terms of the concentrations of species as functions of time and differential equations, is appropriate for reaction systems in aqueous solutions [62,63]. Deterministic differential equation models have given satisfactory predictions for well mixed macroscopic chemical reaction systems. One of the most celebrated examples is the Oregonator: the mathematical theory for the Belousov-Zhabotinsky reactions [64] which display sustained oscillations in a test tube. For a recent study see [28,29].

In recent years, due to the technological advances in optical imaging, single cell analysis, and green fluorescence proteins, experimental observations of biochemical dynamics inside single living cells have become increasingly quantitative [65]. Mathematical modeling of biochemical reaction systems in a living cell requires a different approach. Chemical systems inside a cell, especially those of signaling networks involving transcription regulation, protein phosphorylation and GTPases, often involve a small number of molecules of one or more of the reactants [9,21,66,67]. Such dynamics are usually nonlinear and stochastic, exhibiting random fluctuations. Thus, the traditional method of ordinary differential equations is inappropriate. The fluctuations in the number of molecules, often called “intrinsic noise”, have been shown to have biological significance and contribute to the function of the system [41,68].

Reaction kinetics of this kind are more realistically described by stochastic models that emphasize the discrete nature of molecular reactions and the randomness of their occurrences [61]. The chemical master equation is a class of discrete-state, continuous-time Markov jump processes, known as multi-dimensional birth-death processes in probability theory [69]. Master equation is the widely used name in the physics literature [70]. In a jump process, the chemical reactions are characterized in terms of the stochastic copy numbers of the various dynamic chemical species, which differs from the traditional concetrations by a trivial volume *V* of the reaction system. Reactions occur at random times with exponential distribution. The expected value for the waiting period between each reaction is determined by the number of copies of each species. The differential equation models based on the Law of Mass Action should be thought of as the infinite volume limit of the Markov jump process, known as the thermodynamic limit in statistical physics. As we have seen, the volume *V* is critical to many phenomena which appear only in small, mesoscopic biochemical reaction systems, and thus stochastic kinetic models in theory.

The master equation approach to chemical reactions began in the 1930’s with the work of M.A. Leontovich [71]. Independently, it carried out by A.J.F. Siegert, M. Kac, M. Delbrück, A. Renyi, M. Lax and D.A. McQuarrie, among many others. Comprehensive reviews can be found in [42,61,72,73]. The chemical master equation (CME), first studied by Delbrück in 1940 [74], has become the leading mathematical theory for modeling mesoscopic nonlinear chemical reaction systems with small volume on the order of that of a living cell [8].

From a statistical mechanics point of view, each possible combination of the numbers of the chemical species defines a state of the system. The CME provides the evolution equation of the joint probability distribution function over all system states. In open chemical systems, *i.e.*, where energy is added from an outside source, the number of system states is often infinite, leading to an infinite, coupled system of differential equations for the CME. An analytic solution to the CME for stochastic, open unimolecular reaction networks can be found in [75]. It is not possible, in general, to obtain an analytic solution for an open, non-unimolecular reaction system. However, the “steady state” solution to the master equation (also known as the stationary probability distribution) is generally unique [70] and may be algorithmically computed [76].

Continuous, diffusion approximations (also known as Fokker-Planck approximations) to the master equation were first developed by Van Kampen [77] and shown by Kurtz [78,79] to match the solution to the master equation in the thermodynamic limit for finite time. Because of the “finite time”, the stationary solution at infinite time for the Fokker-Planck equation is often not an acceptable approximation for the stationary solution of the CME. This gives rise to Keizer’s paradox. Fokker-Planck equations describe the probability distribution functions of continuous random movements known as stochastic differential equations (SDE). Approximating stochastic jump processes by diffusion processes with continuous fluctuations, however, is a delicate problem [80,81]. The delicate issue in mathematical term has to do with exchanging the limits for a large number of molecules and for a long time [82]. This limit exchange can lead to disagreements between discrete and continuous stochastic models [36].

The same issue of exchanging limits is present also between a stochastic jump process and the deterministic model. It is intimately related to the time scales for “down-hill dynamics” and “up-hill dynamics” and how their dependence upon the system size *V* [43]. Note that for sufficiently large *V*, the stochastic trajectory is close to the deterministic dynamics. However, there is no deterministic counterpart for stochastic “barrier-crossing” trajectory that moves agains the deterministic vector field. A transition between stable attractors is impossible in a deterministic system, but occurs with probability 1 in stochastic dynamics, albeit with exponentially long time ~ *e^cV^* [44].

Kurtz carried out rigorous studies on the relation between the stochastic theory of chemical kinetics and its deterministic counterpart [83,84]. It has been shown that in the thermodynamic limit of *V* → ∞, the CME becomes the expected deterministic ordinary differential equation (ODE) for finite time. Furthermore, solutions with given initial values to the CME approach the respective solutions to the ODE [83]. In light of this, there can still be disagreement in the steady state (*i.e.*, infinite time limit) solutions, an issue extensively revisited recently by Vellela and Qian [36,43].

Stochastic simulations of complex chemical reaction systems were carried out as early as the 1970’s [85,86]. Current software packages used for the simulation of biochemical reactions commonly make use of algorithms based on the influential work of Gillespie [8,10,87]. Microscopic particle simulations have validated the master equation as the most accurate description of a reactive process in aqueous solution [81,88]; see [89] for an up-to-date review. The CME provides the equation for the time-dependent joint probabilities of the number of molecules while the Gillespie algorithm gives the stochastic trajectories. They correspond to Fokker-Planck equation and stochastic differential equation (SDE) for diffusion processes.

In the environment of a living cell, biochemical systems are operating under a driven condition, widely called an “open system” [12,19,20,90]. There is a material and/or energy flux, from the outside, going through the system [91,92]. Such molecular systems no long obey the traditional theory of equilibrium thermodynamics. A closed molecular system tends to a thermal, chemical equilibrium, which is unique and in which each and every reaction has zero flux [93]. This is known as Lewis’ principle of detailed balance [18]. Under equilibrium conditions, the ODE model based on the Law of Mass Action contains a unique, globally attracting equilibrium (fixed point). Accordingly, the stationary solution to the CME is a multi-Poisson distribution whose peak is located over the ODE fixed point [49].

The nonequilibrium theory for nonlinear biochemical reactions allows the possibility of multiple steady states, and nonzero steady state flux and a nonzero entropy production rate [19,20]. Recent developments in the area of fluctuation theorem [94,95] have illustrated the importance of entropy production and its relationship to the irreversible nature of a system. How is the entropy production rate related to functions of biochemical reaction networks? A correlation has been suggested between entropy production (or “dissipation cost”) and the robustness of a network [96,97]. A more quantitative, if any, relationship between the entropy production rate and the dynamics of a nonequilibrium steady state is yet to be developed.

The essential difference between deterministic and stochastic models is the permanence of fixed points. According to the theory of ordinary differential equation, once the system reaches a fixed point (or an attractor), it must remain there for all time. Systems with stochasticity, however, can have trajectories being pushed away from attracting fixed points by random fluctuations. Since the noise is ever-present, it can eventually push the system out of the basin of attraction of one fixed point (attractor) and into that of another. Fixed points are no longer stationary for all time; they are only temporary, or “quasistationary” [98]. The amount of time the system spends at (or very near) a fixed point increases exponentially with the system volume. This agrees with ODE dynamics in the thermodynamic limit. However, this quasistationary behavior plays an important role at the cellular level in the “cellular evolutionary time scale” [45].

In order to systematically understand the mesoscopic cellular biochemical dynamics, this review discussed the simplest problem that is interesting: a one dimensional system with two fixed points. The systems with only one fixed point are trivial since deterministic and stochastic models are in complete agreement when there is a unique steady state [88]; the linear differential equation corresponds to a Poisson distribution in the CME [75]. The case of two fixed points, one stable and one unstable, is studied through an autocatalytic reaction first introduced by Keizer [42]. The ODE representation takes the form of a classic example in population dynamics, the logistic equation. Through this simple system, one understands the issues in the steady state predictions of the ODE and CME models [43]. This example introduces the notion of a quasistationary fixed point and a spectral analysis reveals the multiple time scales involved in the master equation formulation.

Logically, the next step is a one dimensional system with three fixed points, two stable with one unstable point between them [36,47]. For this, we use a reversible, trimolecular reaction known as Schlögl’s model. This is the first case in which bistable behavior is possible, occurring through a saddle-node bifurcation. Again, the CME allows for new possibilities such as switching between the stable fixed points and a nonequilibrium phase transition in the steady state distribution function [45,51]. Because this model is fully reversible, one is also able to study thermodynamic quantities such as the chemical potential and entropy production rate and to illustrate the nonequilibrium physics [99]. The dynamics of this system serves as a representative example for all systems with multiple stable fixed points.

Once the theory has been established for one dimensional systems with a single dynamic biochemical species, we turn our attention to planar systems with two dynamical species [54]. Here, oscillations become possible in the form of spiral nodes and limit cycles in ODE models. We explore the open question of how to define and quantify stochastic oscillations. We suggest a new method for locating oscillations in the presence of noise by extending the idea of the Poincaré return map to stochastic systems. A reversible extension of Schankenberg’s model for chemical kinetic oscillation is used to illustrate this new idea. The oscillation is represented by a rotational random walk.

In all these studies one encounters the presence of a time scale that grows exponentially with the sysetm’s volume *V*. Dynamics operating on this “cellular evolution time scale” are lost in the infinite volume limit of the ODE model. To study the stability of a stochastic attractor, one must consider the chemical reactions systems in terms of the chemical master equation (CME). The ODE formulation, however, is valuable as a way to estimate the presence and location of the critical points in the landscape of the probability steady state distribution of the CME [100,101].

In summary, one of the most important insights from the CME study of biochemical reaction systems in a small, cellular volume is the realization of the *cellular evolution time scale* and the associated stochastic attractors which might indeed be the emergent cellular epigenetic states. The dynamics on this time scale is stochastic; it is completely missing from the traditional ODE theory of biochemical reaction networks. In the CME theory, deterministic fixed points become stochastic attractors [100,101]. They are the emergent properties of a complex, nonlinear biochemical network. The transitions among the emegent stochastic attractors constitute the proper cellular dynamics [102,103].

## Figures and Tables

**Figure 6:**
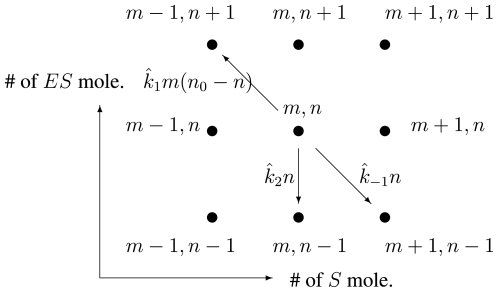
The chemical master equation *graph* for the stochastic Michaelis-Menten enzyme kinetics in (46). The graph only shows all the transitions associated with “leaving” the state (*m*, *n*). What is not shown are the transitions: (*m*−1, *n*+1) → (*m*, *n*) with rate *k̂*_−1_(*n*+1); (*m* + 1, *n* −1) → (*m*, *n*) with rate *k̂*_1_(*m* + 1)(*n*_0_ −*n* + 1); and (*m*, *n* + 1) → (*m*, *n*) with rate *k̂*_2_(*n* + 1). They are all associated with “into” the state (*m*, *n*).

**Figure 7:**
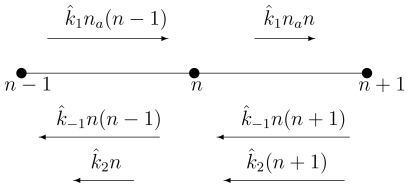
The chemical master equation *graph* shows the probability change in state *P_n_*, where *n_a_* is the number of *A* molecules.

**Figure 8:**
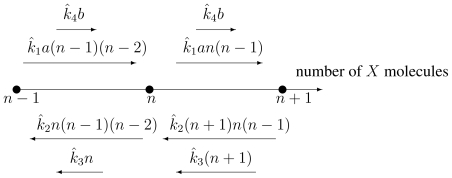
The chemical master equation *graph* for the Schlögl model in Equation (50) shows the rates of probability changes into and leaving state *n. a* and *b* are concentrations of *A* and *B*. The relations between the *k̂*’s and *k*’s in Equation (50) are given in Equation (53).

**Figure 9:**
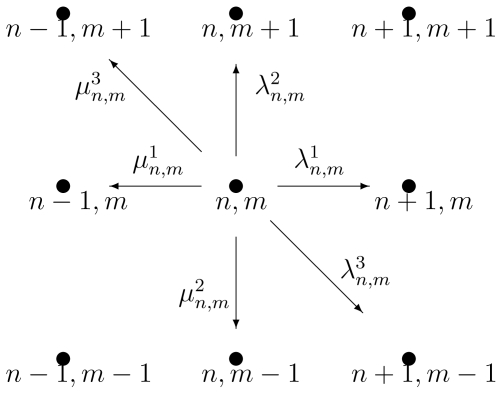
The chemical master equation *graph* showing possible paths *away* from state (*n,m*), with birth rates λ*_n,m_^i^* and death rates *μ_n,m_^i^*. Note that there will be a corresponding reverse path into state (*n,m*) for each of these arrows.

## Appendix: The Chemical Master Equation for Systems of Biochemical Networks

### A1. Michaelis-Menten model

The canonical MM kinetic scheme is

(46) $$E+S\overset{k_{1}}{\underset{k_{-1}}{⇌}}ES\overset{k_{2}}{\to }E+P$$

Let *m* and *n* be the numbers of *S* and *ES* respectively. Assume the total number of enzyme is *n*_0_. Then, the corresponding *chemical master equation graph*, shown below, details the changes in the “state” of the system, (*m*, *n*), due to the three reactions in the Equation (46). The graph in Figure 6 helps one to write the chemical master equation in (2). The three “leaving” terms are negative in Equation (2), and the three “into” terms are positive in Equation (2). The *k̂*’s in the graph is related to the *k*’s in the Equation (46) according to Equation (3).

### A2. Keizer’s Model

We are interested in the autocatalytic reaction system

(47) $$A+X\overset{k_{1}}{\underset{k_{-1}}{⇌}}2X,X\overset{k_{2}}{\to }B$$

in which the concentrations of *A* and *C* are hold constant. This is a modified version of an example originally discussed by J. Keizer in his book [42], where he assumed *k*_−1_ = 0. Let *n*(*t*) be the stochastic number of *X* molecule at time *t*. It is then related to the concentration *x* by *n* = *xV*, where *V* is the volume of the system. This volume parameter *V* appears implicitly in the CME. For example, in the deterministic model, reaction rates *k*_1_ and *k*_−1_ have units of [volume][time]^−1^, and *k*_2_ has units of [time]^−1^. The reaction rates in the stochastic model are related to these rates by

(48) $${\hat{k}}_{1}=k_{1}/V,\;{\hat{k}}_{-1}=k_{-1}/V,\;{\hat{k}}_{2}=k_{2}$$

These reaction rates are scaled such that the units agree in the master equation (see Equation 49 below).

Figure 7 shows how the probability of each state *n* is affected by the three reactions in Equation (47). The change in the probability of each state, $\frac{d}{dt}p_{n}(t)$ is the sum of the changes due to each of the reactions. Thus, the CME is the system of equations:

(49a) $$\frac{dp_{o}}{dt}={\hat{k}}_{2}p_{1}$$

(49b) $$\begin{array}{l} \frac{dp_{n}}{dt}={\hat{k}}_{1}n_{a}(n-1)p_{n-1}+({\hat{k}}_{-1}n(n+1)+{\hat{k}}_{2}(n+1))p_{n+1}\\ -({\hat{k}}_{-1}n(n-1)+{\hat{k}}_{1}n_{a}n+{\hat{k}}_{2}n)p_{n}\\ \vdots \end{array}$$

where *n**_a_* represents the number of *A* molecules, which does not change in the model.

### A3. Schlögl Model

The canonical Schlögl model for chemical bistability is [46]

(50) $$A+2X\overset{k_{1}}{\underset{k_{2}}{⇌}}3X,\;\;\;X\overset{k_{3}}{\underset{k_{4}}{⇌}}B$$

Following the *chemical master equation graph* in Figure 8, we have the CME for the probability of having *n* number of *X* at time *t*, *p**_n_*(*t*) = Pr{*n**_X_*(*t*) = *n*}:

(51) $$\frac{d}{dt}p_{n}(t)=\lambda_{n-1}p_{n-1}-(\lambda_{n}+\mu_{n})p_{n}+\mu_{n+1}p_{n+1}\;\;\;(n\geq 0)$$

where

(52) $$\lambda_{n}={\hat{k}}_{1}an(n-1)+{\hat{k}}_{4}b,\;\;\;\mu_{n}={\hat{k}}_{2}n(n-1)(n-2)+{\hat{k}}_{3}n$$

and

(53) $${\hat{k}}_{1}=\frac{k_{1}}{V},\;{\hat{k}}_{2}=\frac{k_{2}}{V^{2}},\;{\hat{k}}_{3}=k_{3},\;{\hat{k}}_{4}=k_{4}V$$

#### A3.1. Stationary Distribution: Steady State and Equilibrium

By setting the right-hand-side of Equation (51) to zero, one solves the steady state distribution

(54) $$p_{n}^{ss}=C\prod_{i=0}^{n-1}\frac{\lambda_{i}}{\mu_{i+1}}=C\prod_{i=0}^{n-1}\frac{{\hat{k}}_{1}ai(i-1)+{\hat{k}}_{4}b}{{\hat{k}}_{2}(i+1)i(i-1)+{\hat{k}}_{3}(i+1)}$$

where *C* is a normalization factor.

We now show a very interesting and important property of the *p**_n_**^ss^*, when the concetrations of *A* and *B* are at equilibrium: *b*/*a* = *k*_1_*k*_3_/(*k*_2_*k*_4_) = *k̂*_1_*k̂*_3_/(*k̂*_2_*k̂*_4_). Substituting this relation into Equation (54), we have the equilibrium distribution

(55) $$p_{n}^{eq}=C\prod_{i=0}^{n-1}\frac{{\hat{k}}_{1}a}{{\hat{k}}_{2}(i+1)}=\frac{1}{n!}{\left(\frac{{\hat{k}}_{1}a}{{\hat{k}}_{2}}\right)}^{n}e^{-{\hat{k}}_{1}a/{\hat{k}}_{2}}$$

This is a Poisson distribution with the mean number of *X* being $\frac{{\hat{k}}_{1}a}{{\hat{k}}_{2}}=\frac{k_{1}aV}{k_{2}}$. That is the equilibrium concentration of *X* being $\frac{k_{1}}{k_{2}}a$. The Poisson distribution has only a single peak.

### A4. Schnakenberg Model

We are now interested in the nonlinear chemical reaction system, the reversible Schnakenberg model, in a mesoscopic volume *V* :

(56) $$X\overset{k_{1}}{\underset{k_{-1}}{⇌}}A,\;\;\;B\overset{k_{2}}{\underset{k_{-2}}{⇌}}Y,\;\;\;2X+Y\overset{k_{3}}{\underset{k_{-3}}{⇌}}3X$$

Consider the function *p**_n,m_*(*t*), the probability that there are *n X* molecules and *m Y* molecules at time *t*. The six reactions (three forward and three backward) in the reversible Schnakenberg model in Equation (56) define six ways to move on the lattice of possible states, *i.e.*, the (*n*,*m*) lattice, ℤ^+^ × ℤ^+^ (see Figure 9). The chemical master equation (CME) is a doubly infinite set of ODEs:

(57) $$\begin{array}{l} \frac{dp_{n,m}(t)}{dt}=\lambda_{n-1,m}^{1}p_{n-1,m}+\lambda_{n,m-1}^{2}p_{n,m-1}+\lambda_{n-1,m+1}^{2}p_{n-1,m+1}\\ +\mu_{n+1,m}^{1}p_{n+1,m}+\mu_{n,m+1}^{2}p_{n,m+1}+\mu_{n+1,m-1}^{3}p_{n+1,m-1}\\ -(\lambda_{n,m}^{1}+\lambda_{n,m}^{2}+\lambda_{n,m}^{3}+\mu_{n,m}^{1}+\mu_{n,m}^{2}+\mu_{n,m}^{3})p_{n,m} \end{array}$$

for *n* = 0 . . . ∞, *m* = 0 . . . ∞. The birth and death rates, λ*_n,m_**^i^* and *μ**_n,m_**^i^* respectively, for the three equations are

(58) $$\lambda_{n,m}^{1}=k_{-1}n_{a},\;\;\;\mu_{n,m}^{1}=k_{1}n$$

(59) $$\lambda_{n,m}^{2}=k_{2}n_{b},\;\;\;\mu_{n,m}^{2}=k_{-2}m$$

(60) $$\lambda_{n,m}^{3}=\frac{k_{3}}{V^{2}}n(n-1)m,\;\;\;\mu_{n,m}^{3}=\frac{k_{-3}}{V^{2}}n(n-1)(n-2)$$

The factor of 1/*V*^2^ in λ*_n,m_*^3^ and *μ**_n,m_*^3^ accounts for the fact that the third reaction is trimolecular, and thus *k*_3_ and *k*_−3_ have units of *V*^2^/*t*. Since the first and second reactions are unimolecular, the remaining rates have units of 1/*t* and do not need scaling when used in the CME.

Because the CME is a set of linear ODEs, there will be a unique steady state to which the system tends, the probability steady state, *p**^ss^*(*n,m*). Once the system reaches its steady state, the total probability flow into each point, (*n,m*), will equal the total probability flow out of that point. Solving for *p**^ss^*(*n,m*) involves setting each of the equations in Equation 57 equal to zero and solving a very large linear system. Cao and Liang have recently developed a method for computing the probability steady state for molecular networks in general [76]. Their method is an algorithm which enumerates the state space and solves the corresponding linear system and is optimal in both storage and time complexity.
